# Supplementary material for: scClassify: sample size estimation and multiscale classification of cells using single and multiple reference
Source: Mol Syst Biol. 2020 Jun 22;16(6):e9389. doi: 10.15252/msb.20199389 (PMC7306901; doi:10.15252/msb.20199389)
Supplement: Supplementary file 1 — Appendix [file MSB-16-e9389-s001.pdf]

Table of Content

List of Figures

Appendix Fig S1 tSNE plots of the Muraro et al. dataset from the human pancreas data collection: tSNE plots are color-coded by their original label (panel (1,1)) or predicted cell types from 15 different methods, scClassify, SingleR, moana, singlecellNet, ACTINN, CHETAH, scID, Garnett (marker), Garnett (DE), scmap-cell, scmap-cluster, scPred, SVMreject, CatSLe, which all used the Wang et al. dataset as the reference dataset (see Supp Table 1 for details). Under default settings, scClassify is able to correctly classify most cells with an accuracy rate of greater than 95%. None of the methods were fine-tuned specific on the training or test datasets. 3

Appendix Fig S2 Computation time and memory benchmarking results: A. The computation time of different methods against the number of cells in reference, ranging from 100 to 30000, where the number of cells in query data is fixed to be 2000. B. The computation time of different methods against the number of cells in query, ranging from 100 to 30000, where the number of cells in reference data is fixed to be 2000. C. The computation time of different methods against the number of cell types in the reference and query dataset, ranging from 4 to 12. D. The memory requirement of different methods against the number of cells in reference, ranging from 100 to 30000, where the number of cells in query data is fixed to be 2000. 4

Appendix Fig S3 Sensitivity analysis results for the maximum number of children per branch node in HOPACH tree: Each box indicates the classification accuracy with different maximum number of children per branch node, ranging from 3 to 11, using 30 training and test data pairs from the Pancreas data collection, with 16 easy cases (left panel) and 14 hard cases (right panel). 5

Appendix Fig S4 Simulation results for sample size calculation. A. A 4 by 1 panel indicating the accuracy rate of the simulation results using SymSim [19] by estimating parameters from PBMC10k dataset. Each of four plots indicates accuracy rate with different degrees of within cell type heterogeneity (0.2, 0.4, 0.6, and 0.8), colored coded by five different sequencing depth (30000, 80000, 160000, 300000, 500000). The horizontal axis shows capture efficiencies ranged from 0.001 to 0.1, and y-axis indicates the accuracy rate. B. A 4 by 1 panel of fitted learning curves of the simulation results, where each plot indicates accuracy rate of different degrees of within cell type heterogeneity (0.2, 0.4, 0.6, and 0.8), colored coded by different capture efficiency (0.001, 0.005, 0.01, 0.2, 0.05, and 0.1). X-axis indicates the sample size (N) of the reference set, and y-axis indicates the accuracy rate. 6

Appendix Fig S5 Downsampling of the PBMC10k data using DECENT's beta-binomial capture model [18]. Sample size calculation of down sampling. The left panel indicates the accuracy rate generated by repeating the training and testing procedure 50 times with varying size of the reference data and probabilities for down-sampling. The right panel displays the fitted learning curves based on the mean accuracy rate of the left panel. Both boxplots and lines are colored by probability of down-sampling (0.2, 0.5, 0.8 and 1). The top panel shows the results from the cell type predictions at the top level of the cell type tree, and the bottom 7

Appendix Fig S6 Tabula Muris cell type tree. A cell type tree generated using the hierarchical ordered partitioning and collapsing hybrid (HOPACH) algorithm and the Tabula Muris FACS data as the reference dataset [13]. 8

Appendix Fig S7 A. A 1 by 3 panel of tSNE plots of the Tasic et al. dataset (2016) from the neuronal data collection, where data points are color coded by original cell types given in Tasic et al, 2016 (left panel) [15], the scClassify predicted cell types generated using Tasic et al. (2018) as the reference dataset (middle panel) and the scClassify predicted cell types generated using Hrvatin et al. as the reference dataset (right panel). B. A 1 by 3 panel of tSNE plots of Tasic et al. (2018) from the neuronal data collection color coded by the original cell types given in Tasic et al, 2018 (left panel) [16], the scClassify predicted cell types generated using Tasic et al. (2016) as the reference dataset (middle panel) and the scClassify predicted cell types generated using Hrvatin et al. as the reference dataset (right panel). C. A 1 by 3 panel of tSNE plots of Hrvatin et al. from the neuronal data collection color coded by the original label (left panel) [5], the scClassify predicted cell types generated using Tasic et al. (2016) as the reference dataset (middle panel) and the scClassify predicted cell types generated using Tasic et al. (2018) as the reference dataset (right panel). The accuracy rate of common cell types in reference and query data are 92.3% (Tasic 2016) and 97.3% (Tasic 2018) when the Hrvatin data was used as reference; 95.8% (Tasic 2018) and 90.6% (Hrvatin) when the Tasic 2016 data was used; and 95.6% (Tasic 2016) and 95.3% (Hrvatin) when the Tasic 2018 data was used. 9

Appendix Fig S8 A. A 1 by 2 panel heatmaps of data in Appendix Fig S7 a comparing the cell types from the original cell types given in Tasic (2016) (rows) against scClassify predicted cell types (columns) generated using either the Tasic (2018) (left panel) or the Hrvatin et al. (right panel) as reference dataset. The squares are colored by the percentage of cells of a certain Tasic (2016) cell type. (B-C) as above Appendix Fig S7 B and C 10

List of Tables

Appendix Table S1 Current supervised learning methods for cell-type identification. 11

Appendix Table S2 Similarity metrics we considered in this study to measure dissimilarity between these two cells. For simplicity in what follows, we identify cells with their (cell-type specific) gene expression vectors. Let $\mathbf{x}, \mathbf{y} \in \mathcal{R}^m$ be cells with m gene expression values selected from the reference and query/test datasets, respectively. Note that the cosine and Jaccard distances are calculated using the *proxy* package [10]. scClassify uses Pearson's correlation by default. 12

Figure S1.pdf

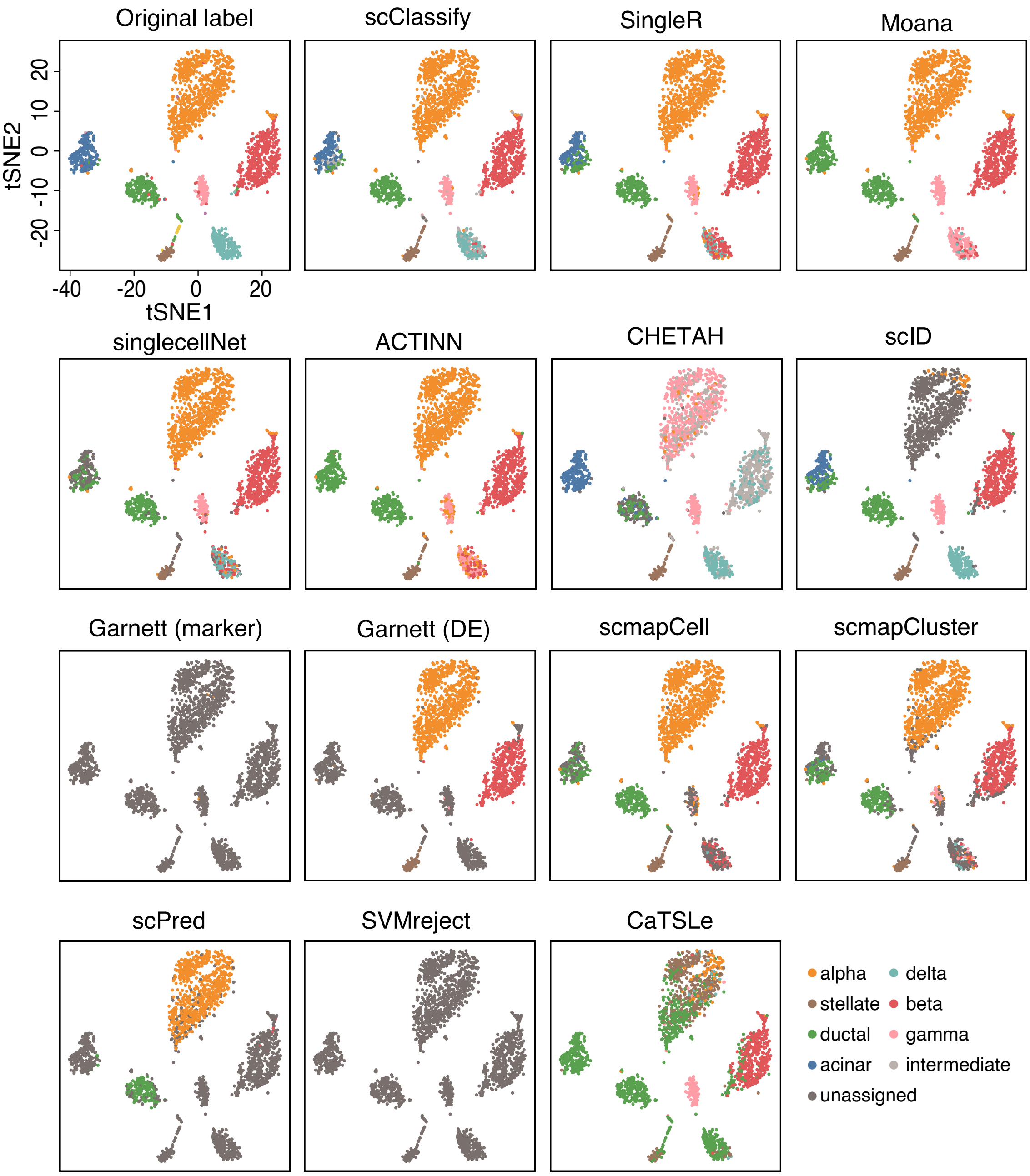

Appendix Fig S1: tSNE plots of the Muraro et al. dataset from the human pancreas data collection: tSNE plots are color-coded by their original label (panel (1,1)) or predicted cell types from 15 different methods, scClassify, SingleR, moana, singlecellNet, ACTINN, CHETAH, scID, Garnett (marker), Garnett (DE), scmap-cell, scmap-cluster, scPred, SVMreject, CatSLe, which all used the Wang et al. dataset as the reference dataset (see Supp Table 1 for details). Under default settings, scClassify is able to correctly classify most cells with an accuracy rate of greater than 95%. None of the methods were fine-tuned specific on the training or test datasets.

Figure S2.pdf

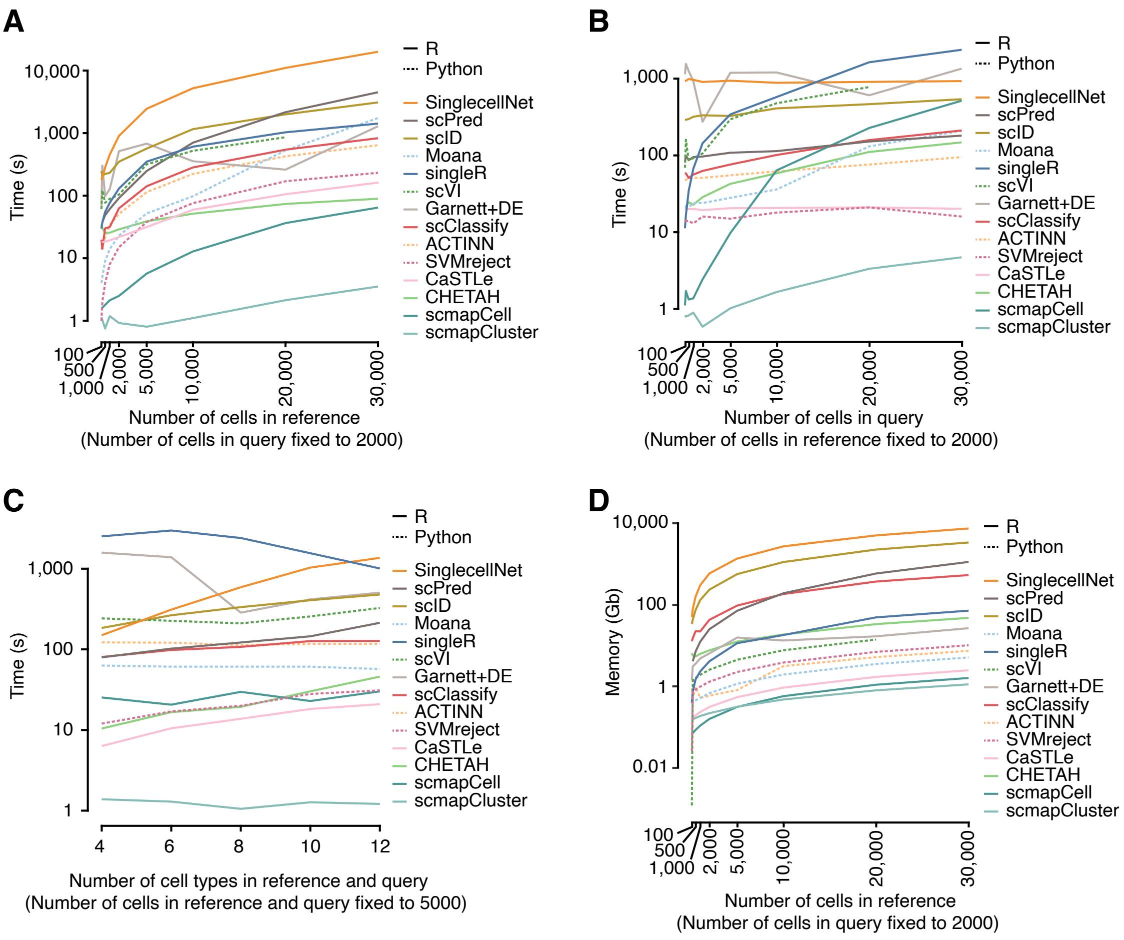

Appendix Fig S2: Computation time and memory benchmarking results: A. The computation time of different methods against the number of cells in reference, ranging from 100 to 30000, where the number of cells in query data is fixed to be 2000. B. The computation time of different methods against the number of cells in query, ranging from 100 to 30000, where the number of cells in reference data is fixed to be 2000. C. The computation time of different methods against the number of cell types in the reference and query dataset, ranging from 4 to 12. D. The memory requirement of different methods against the number of cells in reference, ranging from 100 to 30000, where the number of cells in query data is fixed to be 2000.

Figure S3.pdf

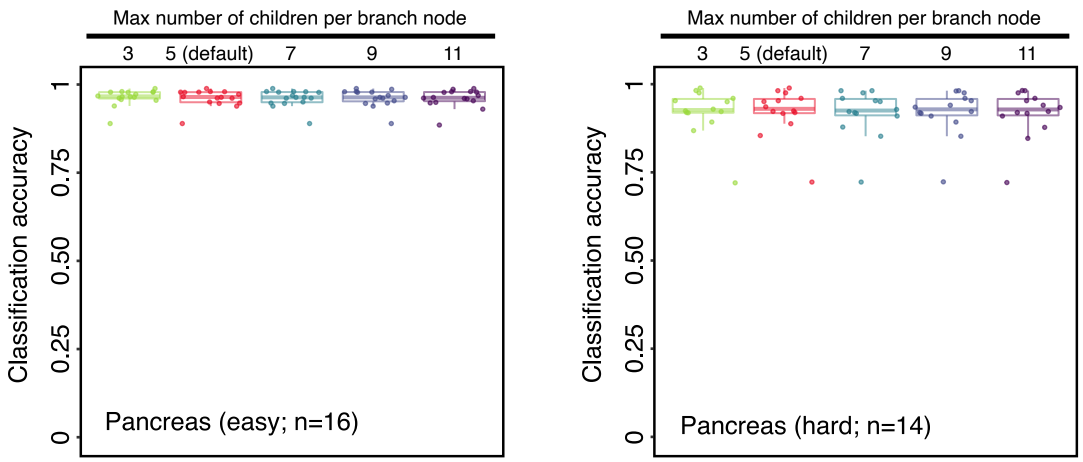

Appendix Fig S3: Sensitivity analysis results for the maximum number of children per branch node in HOPACH tree: Each box indicates the classification accuracy with different maximum number of children per branch node, ranging from 3 to 11, using 30 training and test data pairs from the Pancreas data collection, with 16 easy cases (left panel) and 14 hard cases (right panel).

Figure S4.pdf

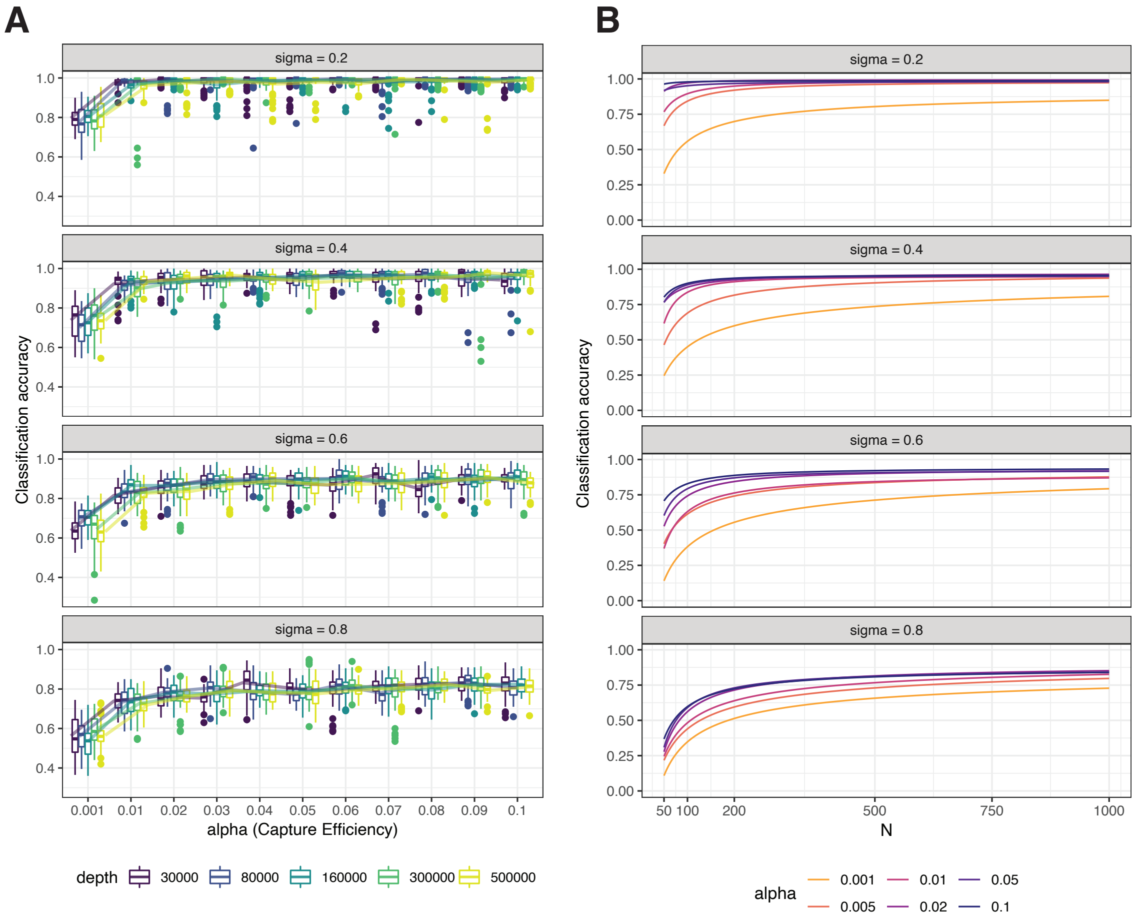

Appendix Fig S4: Simulation results for sample size calculation. A. A 4 by 1 panel indicating the accuracy rate of the simulation results using SymSim [19] by estimating parameters from PBMC10k dataset. Each of four plots indicates accuracy rate with different degrees of within cell type heterogeneity (0.2, 0.4, 0.6, and 0.8), colored coded by five different sequencing depth (30000, 80000, 160000, 300000, 500000). The horizontal axis shows capture efficiencies ranged from 0.001 to 0.1, and y-axis indicates the accuracy rate. B. A 4 by 1 panel of fitted learning curves of the simulation results, where each plot indicates accuracy rate of different degrees of within cell type heterogeneity (0.2, 0.4, 0.6, and 0.8), colored coded by different capture efficiency (0.001, 0.005, 0.01, 0.2, 0.05, and 0.1). X-axis indicates the sample size (N) of the reference set, and y-axis indicates the accuracy rate.

Figure S5.pdf

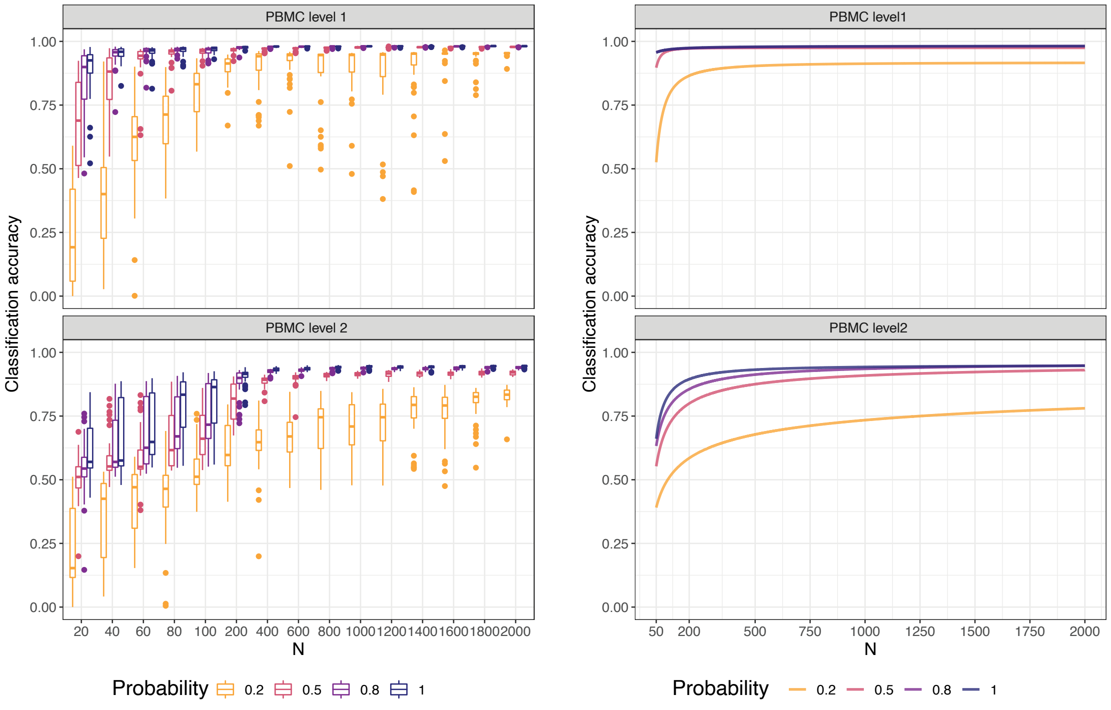

Appendix Fig S5: Downsampling of the PBMC10k data using DECENT's beta-binomial capture model [18]. Sample size calculation of down sampling. The left panel indicates the accuracy rate generated by repeating the training and testing procedure 50 times with varying size of the reference data and probabilities for down-sampling. The right panel displays the fitted learning curves based on the mean accuracy rate of the left panel. Both boxplots and lines are colored by probability of down-sampling (0.2, 0.5, 0.8 and 1). The top panel shows the results from the cell type predictions at the top level of the cell type tree, and the bottom

Figure S6.pdf

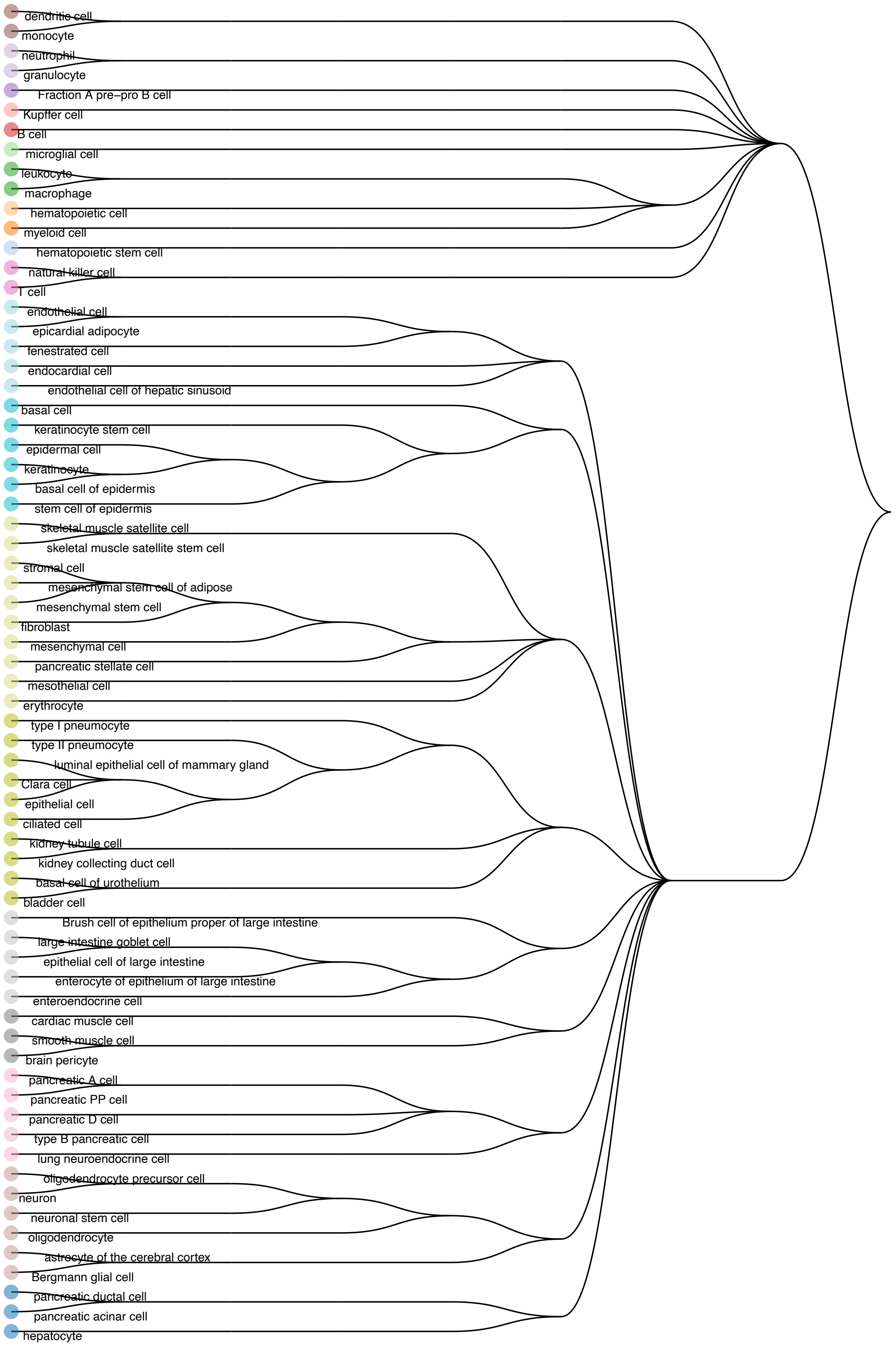

Appendix Fig S6: Tabula Muris cell type tree. A cell type tree generated using the hierarchical ordered partitioning and collapsing hybrid (HOPACH) algorithm and the Tabula Muris FACS data as the reference dataset [13].

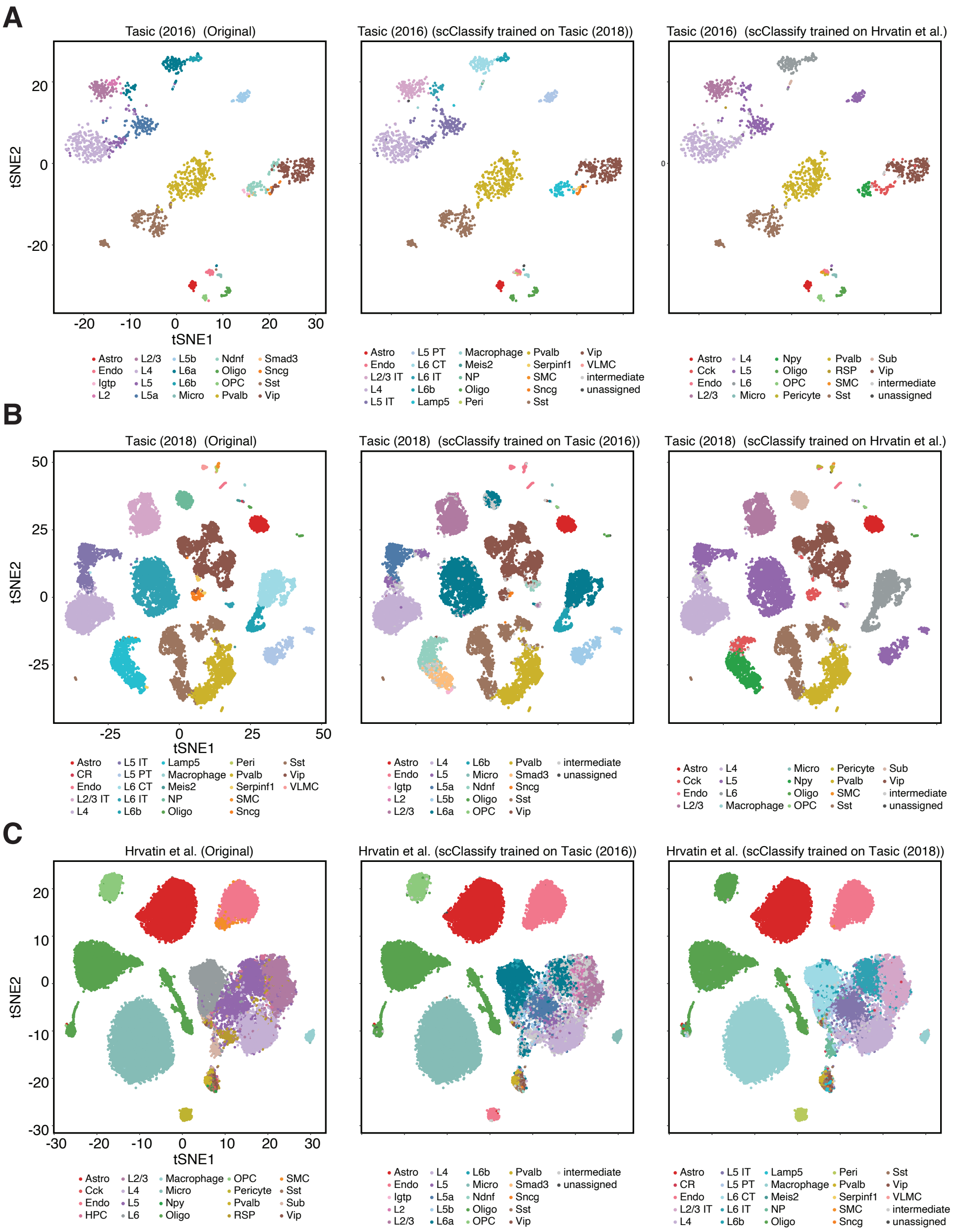

Figure S7.pdf

Appendix Fig S7: A. A 1 by 3 panel of tSNE plots of the Tasic et al. dataset (2016) from the neuronal data collection, where data points are color coded by original cell types given in Tasic et al, 2016 (left panel) [15], the scClassify predicted cell types generated using Tasic et al. (2018) as the reference dataset (middle panel) and the scClassify predicted cell types generated using Hrvatin et al. as the reference dataset (right panel). B. A 1 by 3 panel of tSNE plots of Tasic et al. (2018) from the neuronal data collection color coded by the original cell types given in Tasic et al, 2018 (left panel) [16], the scClassify predicted cell types generated using Tasic et al. (2016) as the reference dataset (middle panel) and the scClassify predicted cell types generated using Hrvatin et al. as the reference dataset (right panel). C. A 1 by 3 panel of tSNE plots of Hrvatin et al. from the neuronal data collection color coded by the original label (left panel) [5], the scClassify predicted cell types generated using Tasic et al. (2016) as the reference dataset (middle panel) and the scClassify predicted cell types generated using Tasic et al. (2018) as the reference dataset (right panel). The accuracy rate of common cell types in reference and query data are 92.3% (Tasic 2016) and 97.3% (Tasic 2018) when the Hrvatin data was used as reference; 95.8% (Tasic 2018) and 90.6% (Hrvatin) when the Tasic 2016 data was used; and 95.6% (Tasic 2016) and 95.3% (Hrvatin) when the Tasic 2018 data was used.

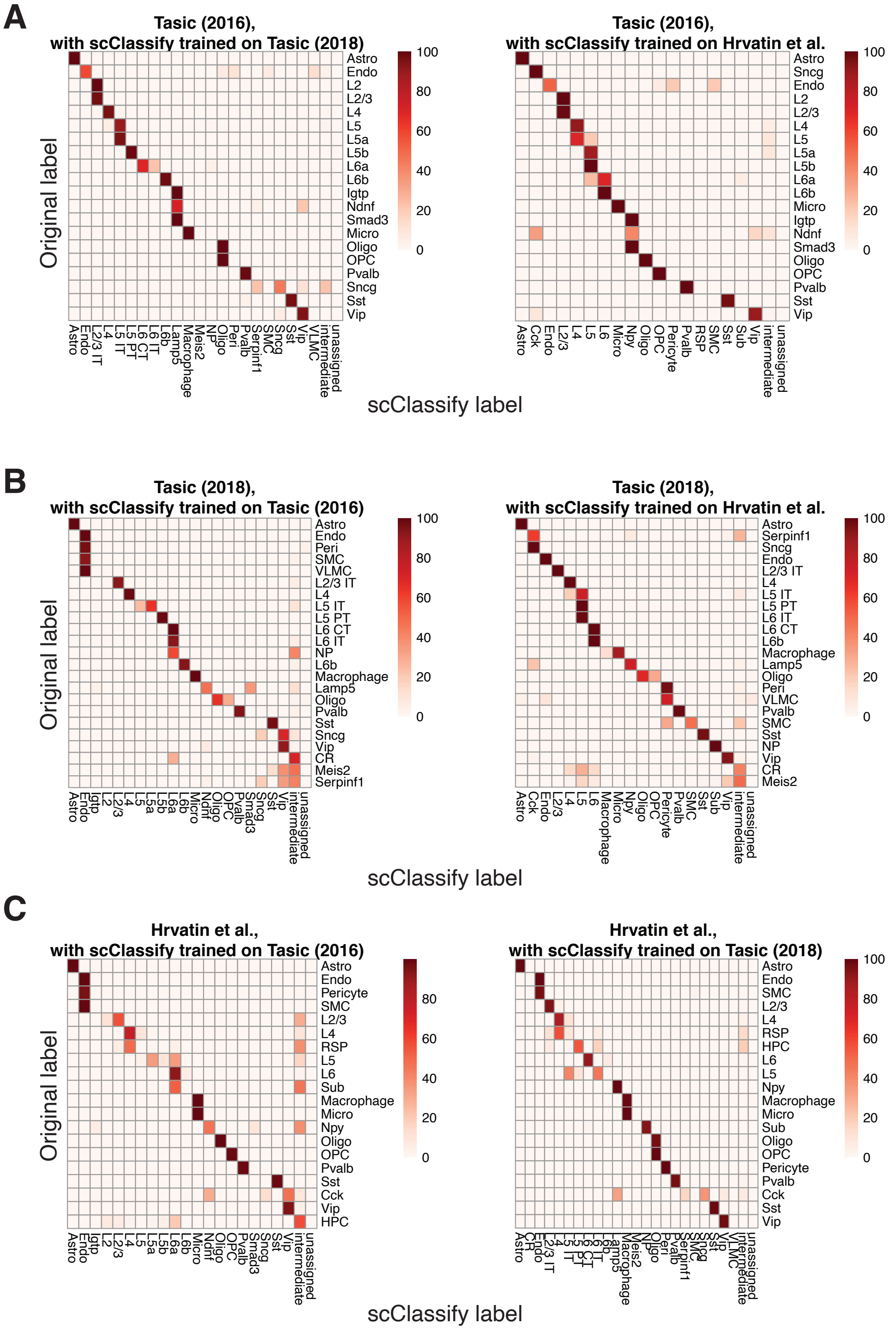

Figure S8.pdf

Appendix Fig S8: A. A 1 by 2 panel heatmaps of data in Appendix Fig S7 a comparing the cell types from the original cell types given in Tasic (2016) (rows) against scClassify predicted cell types (columns) generated using either the Tasic (2018) (left panel) or the Hrvatin et al. (right panel) as reference dataset. The squares are colored by the percentage of cells of a certain Tasic (2016) cell type. (B-C) as above Appendix Fig S7 B and C

Appendix Table S1: Current supervised learning methods for cell-type identification.

Method	Version	Unassigned	Intermediate	Method	Prior Knowledge	Input	Allow multiple reference	Quantify uncertainty	Reference
ACTINN	c3dd085 (Github)	×	×	Neural Network	×	raw count	×	Proability	[9]
CHETAH	1.1.2	✓	✓	Correlation to training set, Hierachical classifciation	×	normalised count	×	Confidence score	[4]
CaSTLe	258b278 (Github)	×	×	XGBoost	×	normalised count	×	Probability	[7]
Garnett	0.1.4	✓	×	Generalized linear model	✓	raw count	×	×	[12]
SingleR	1.0.1	×	×	Correlation to training set	×	input: raw or normalised; reference : normalised	✓	Correlation	[2]
Moana	0.1.1	×	×	SVM with linear kernel	✓	Did not specify	×	×	[17]
scID	0.0.0.9000	✓	×	LDA	×	raw or library-depth normalization	×	Probability	[3]
scPred	0.0.0.9000	✓	×	SVM with a radial kernel	×	raw count or normalised count	×	Probability	[1]
scVI	0.3.0	×	×	Neural Network	×	raw count	×	Probability	[8]
scmap	1.1.6	✓	×	correlation based kNN	×	normalised count	✓	Probability	[6]
SingleCellNet	0.1.0	×	×	Random forest	×	raw count	×	Correlation	[14]
SVMreject	0.22.2	✓	×	SVM with a linear kernel	×	Did not specify	✓	Probability	[11]
scClassify	0.2.0	✓	✓	Ensemble hierachical classifciation, correlation based kNN	×	log normalised count	✓	Correlation and weighted score	**this study**

Appendix Table S2: Similarity metrics we considered in this study to measure dissimilarity between these two cells. For simplicity in what follows, we identify cells with their (cell-type specific) gene expression vectors. Let $\mathbf{x}, \mathbf{y} \in \mathcal{R}^m$ be cells with m gene expression values selected from the reference and query/test datasets, respectively. Note that the cosine and Jaccard distances are calculated using the *proxy* package [10]. scClassify uses Pearson's correlation by default.

	Similarity metrics	Formula
1	Pearson correlation	$$d = 1 - \frac{\sum_{i=1}^{m}(x_i - \bar{x})(y_i - \bar{y})}{\sqrt{\sum_{i=1}^{m}(x_i - \bar{x})^2}\sqrt{\sum_{i=1}^{m}(y_i - \bar{y})^2}}.$$
2	Spearman correlation	$$d = 1 - \frac{\sum_{i=1}^{m}(r_i^x - \bar{r^x})(r_i^y - \bar{r^y})}{\sqrt{\sum_{i=1}^{m}(r_i^x - \bar{r^x})^2}\sqrt{\sum_{i=1}^{m}(r_i^y - \bar{r^y})^2}},$$ where r_i^x, r_i^y denote the rank of the expression value of gene i in cell $\mathbf{x}$, $\mathbf{y}$ respectively; and $\bar{r}$ indicates the the mean rank of expression of the cell.
3	Kendall rank correlation	$$d = 1 - \frac{2}{n(n-1)} \sum_{i<j} \text{sign}(x_i - x_j)\text{sign}(y_i - y_j).$$
4	Cosine distance	$$d = 1 - \frac{\sum_{i=1}^{m} x_i y_i}{\sqrt{\sum_{i=1}^{m} x_i^2}\sqrt{\sum_{i=1}^{m} y_i^2}}.$$
5	Jaccard distance	$$d = 1 - \frac{\lvert A_i \cap A_j \rvert}{\lvert A_i \cup A_j \rvert},$$ where A_i, A_j indicate the set of genes that with expression greater than zero in cell i and cell j.
6	Weighted ranked correlation	$$S_i = \sum_{j=i}^{m} \frac{1}{j},$$ where i is the rank assigned to the i-th largest of the m gene expression values. Here, we are giving higher weight to agreement on the top rankings.

References

[1] Jose Alquicira-Hernandez, Anuja Sathe, Hanlee P Ji, Quan Nguyen, and Joseph E Powell. scPred: accurate supervised method for cell-type classification from single-cell RNA-seq data. *Genome Biology*, 20(1):264, 2019.

[2] Dvir Aran, Agnieszka P Looney, Leqian Liu, Esther Wu, Valerie Fong, Austin Hsu, Suzanna Chak, Ram P Naikawadi, Paul J Wolters, Adam R Abate, et al. Reference-based analysis of lung single-cell sequencing reveals a transitional profibrotic macrophage. *Nature immunology*, 20(2):163, 2019.

[3] Katerina Boufea, Sohan Seth, and Nizar N Batada. scid: Identification of transcriptionally equivalent cell populations across single cell rna-seq data using discriminant analysis. *bioRxiv*, page 470203, 2019.

[4] Jurrian K. de Kanter, Philip Lijnzaad, Tito Candelli, Thanasis Margaritis, and Frank C.P. Holstege. CHETAH: a selective, hierarchical cell type identification method for single-cell RNA sequencing. *Nucleic acids research*, 2019.

[5] Sinisa Hrvatin, Daniel R Hochbaum, M Aurel Nagy, Marcelo Cicconet, Keiramarie Robertson, Lucas Cheadle, Rapolas Zilionis, Alex Ratner, Rebeca Borges-Monroy, Allon M Klein, et al. Single-cell analysis of experience-dependent transcriptomic states in the mouse visual cortex. *Nature neuroscience*, 21(1):120, 2018.

[6] Vladimir Yu Kiselev, Andrew Yiu, and Martin Hemberg. scmap: projection of single-cell rna-seq data across data sets. *Nature methods*, 15(5):359, 2018.

[7] Yuval Lieberman, Lior Rokach, and Tal Shay. Castle–classification of single cells by transfer learning: Harnessing the power of publicly available single cell rna sequencing experiments to annotate new experiments. *PloS One*, 13(10):e0205499, 2018.

[8] Romain Lopez, Jeffrey Regier, Michael B Cole, Michael I Jordan, and Nir Yosef. Deep generative modeling for single-cell transcriptomics. *Nature methods*, 15(12):1053, 2018.

[9] Feiyang Ma and Matteo Pellegrini. Actinn: automated identification of cell types in single cell rna sequencing. *Bioinformatics*, 2019.

[10] David Meyer and Christian Buchta. *proxy: Distance and Similarity Measures*, 2019. R package version 0.4-23.

[11] F. Pedregosa, G. Varoquaux, A. Gramfort, V. Michel, B. Thirion, O. Grisel, M. Blondel, P. Prettenhofer, R. Weiss, V. Dubourg, J. Vanderplas, A. Passos, D. Cournapeau, M. Brucher, M. Perrot, and E. Duchesnay. Scikit-learn: Machine learning in Python. *Journal of Machine Learning Research*, 12:2825–2830, 2011.

[12] Hannah A Pliner, Jay Shendure, and Cole Trapnell. Supervised classification enables rapid annotation of cell atlases. *Nature Methods*, 16(10):983–986, 2019.

[13] Nicholas Schaum, Jim Karkanias, Norma F Neff, Andrew P May, Stephen R Quake, Tony Wyss-Coray, Spyros Darmanis, Joshua Batson, Olga Botvinnik, Michelle B Chen, et al. Single-cell transcriptomics of 20 mouse organs creates a tabula muris: The tabula muris consortium. *Nature*, 562(7727):367, 2018.

[14] Yuqi Tan and Patrick Cahan. Singlecellnet: a computational tool to classify single cell rna-seq data across platforms and across species. *Cell systems*, 9(2):207–213, 2019.

[15] Bosiljka Tasic, Vilas Menon, Thuc Nghi Nguyen, Tae Kyung Kim, Tim Jarsky, Zizhen Yao, Boaz Levi, Lucas T Gray, Staci A Sorensen, Tim Dolbeare, et al. Adult mouse cortical cell taxonomy revealed by single cell transcriptomics. *Nature neuroscience*, 19(2):335, 2016.

[16] Bosiljka Tasic, Zizhen Yao, Lucas T Graybuck, Kimberly A Smith, Thuc Nghi Nguyen, Darren Bertagnolli, Jeff Goldy, Emma Garren, Michael N Economo, Sarada Viswanathan, et al. Shared and distinct transcriptomic cell types across neocortical areas. *Nature*, 563(7729):72, 2018.

[17] Florian Wagner and Itai Yanai. Moana: A robust and scalable cell type classification framework for single-cell rna-seq data. *BioRxiv*, page 456129, 2018.

[18] Chengzhong Ye, Terence P Speed, and Agus Salim. DECENT: differential expression with capture efficiency adjustmeNT for single-cell RNA-seq data. *Bioinformatics*, 2019.

[19] Xiuwei Zhang, Chenling Xu, and Nir Yosef. Simulating multiple faceted variability in single cell rna sequencing. *Nature communications*, 10(1):2611, 2019.
