# Supplementary material for: scClassify: sample size estimation and multiscale classification of cells using single and multiple reference
Source: Mol Syst Biol. 2020 Jun 22;16(6):e9389. doi: 10.15252/msb.20199389 (PMC7306901; doi:10.15252/msb.20199389)
Supplement: Supplementary file 2 — Expanded View Figures PDF [file MSB-16-e9389-s002.pdf]

# Expanded View Figures

**Figure EV1. scClassify evaluation framework and data collections. Related to Fig 1.**

A Evaluation framework used in this study. Predictions are classified into “correctly classified”, “misclassified”, “intermediate” (either correct or incorrect), “incorrectly unassigned”, “incorrectly assigned” or “correctly unassigned”.

B All datasets used in this study, including two collections for benchmarking and five collections for case study in sample size learning and rare cell type identification.

© 2020 The Authors 

**A**

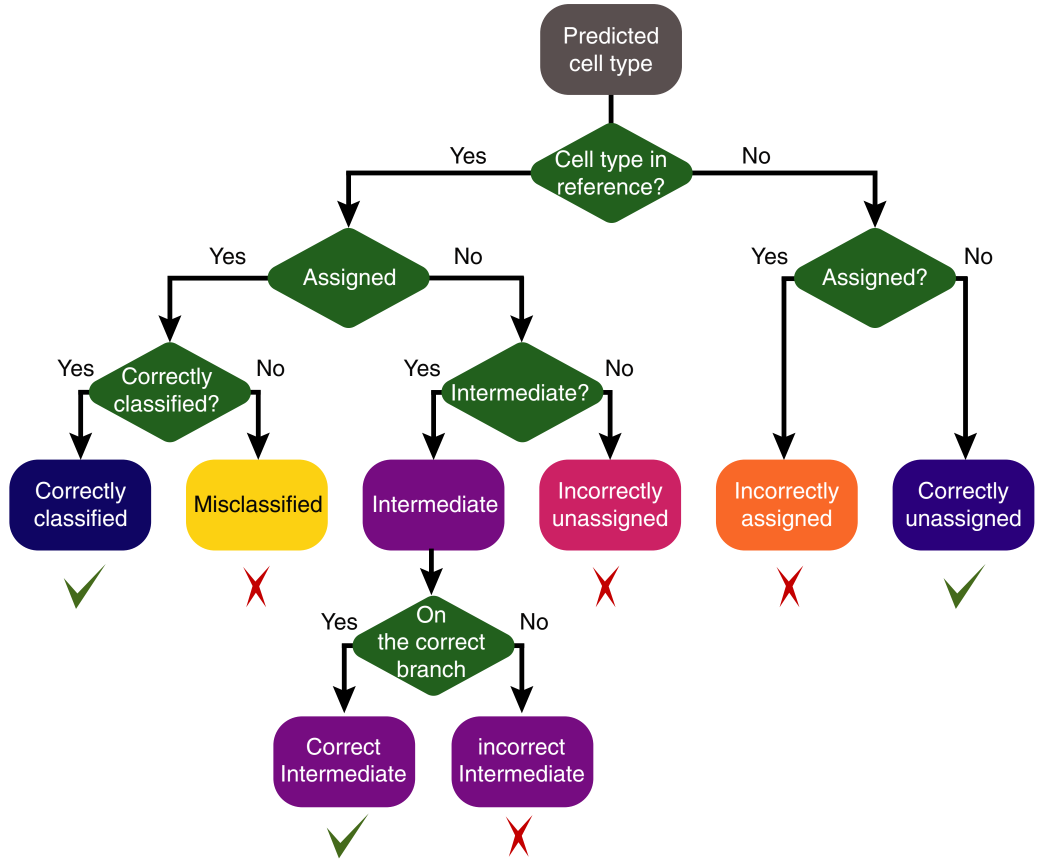


**B**

| | Data collection | Accession | Name | Protocol | Organism | Tissue | # of cell types | # of cells |
|---|---|---|---|---|---|---|---|---|
| Benchmark data collections | Pancreas | GSE81608 | Xin | SMARTer/C1 | Human | Pancreas | 4 | 1474 |
| | | GSE83139 | Wang | SMART-seq | | | 7 | 501 |
| | | GSE86469 | Lawlor | SMARTer/C1 | | | 7 | 617 |
| | | E-MTAB-5061 | Segerstolpe | SMART-seq2 | | | 11 | 2127 |
| | | GSE85241 | Muraro | Cel-seq2 | | | 9 | 2122 |
| | | GSE84133 | Baron | inDrop | | Pancreas Islets | 13 | 8569 |
| | PBMC | NA | Smart-seq | Smart-seq | Human | PBMC | 7 | 526 |
| | | | CEL-seq | CEL-seq | | | 7 | 526 |
| | | | 10x (V2) | 10x (V2) | | | 8 | 3362 |
| | | | 10x (V3) | 10x (V3) | | | 9 | 3222 |
| | | | inDrop | inDrop | | | 9 | 6584 |
| | | | seqWells | seqWells | | | 7 | 6584 |
| | | | Drop-seq | Drop-seq | | | 9 | 3727 |
| Case study data collections | Tabula Muris | GSE109774 | Tabula Muris FACS | FACS | Mouse | Multiple | 68 | 41965 |
| | | | Tabula Muris Microfluidic | Microfluidic | | | 40 | 54439 |
| | Neuronal | GSE71585 | Tasic (2016) | SMARTer/C1 | Mouse | Primary visual cortex | 20 | 1679 |
| | | GSE115746 | Tasic (2018) | SMART-seq2 | | | 23 | 13586 |
| | | GSE102827 | Hrvatin | inDrop | | Visual cortex | 20 | 48266 |
| | 10x PBMC | NA | 10x10k | 10x (V3) | Human | PBMC | 7 | 10753 |
| | Lung | GSE119228 | Cohen | MARS-seq | Mouse | Lung | 22 | 20931 |

**Figure EV1.**


© 2020 The Authors

**Figure EV2. Benchmark results for 16 different methods and sensitivity analysis of hyperparameters of scClassify. Related to Fig 2.**

A Benchmarking results for 16 different methods. Each bar indicates the composition of predicted categories of the average performance in a collection of reference–testing pairs. We divided reference–test pairs into four groups: pancreas (easy), pancreas (hard), PBMC (level 1) and PBMC (level 2).

B Each box indicates the classification accuracy by subsampling 80% of training data, repeated 10 times, using 30 training and test data pairs from the pancreas data collection. The red dots indicate the classification accuracy of using the full training data. Each boxplot ranges from the first to third quartile of classification accuracy with the median as the horizontal line. The lower and higher whiskers of boxplot are extended to the first quartile minus 1.5 interquartile range and the third quartile plus 1.5 interquartile, respectively.

C Each box indicates the classification accuracy with different number of nearest neighbours ($k$ = 5, 10, 15 and 20) to be considered in the weighted $k$NN, using 30 training and test data pairs from the pancreas data collection, with 16 easy cases (top panel) and 14 hard cases (bottom panel). Each boxplot ranges from the first to third quartile of classification accuracy with the median as the horizontal line. The lower and higher whiskers of boxplot are extended to the first quartile minus 1.5 interquartile range and the third quartile plus 1.5 interquartile, respectively.

D Each box indicates the classification accuracy with different correlation thresholds determined, either dynamic or pre-defined (ranging from 0 to 0.8), using 30 training and test data pairs from the pancreas data collection, with 16 easy cases (top panel) and 14 hard cases (bottom panel). Each boxplot ranges from the first to third quartile of classification accuracy with the median as the horizontal line. The lower and higher whiskers of boxplot are extended to the first quartile minus 1.5 interquartile range and the third quartile plus 1.5 interquartile, respectively.

© 2020 The Authors

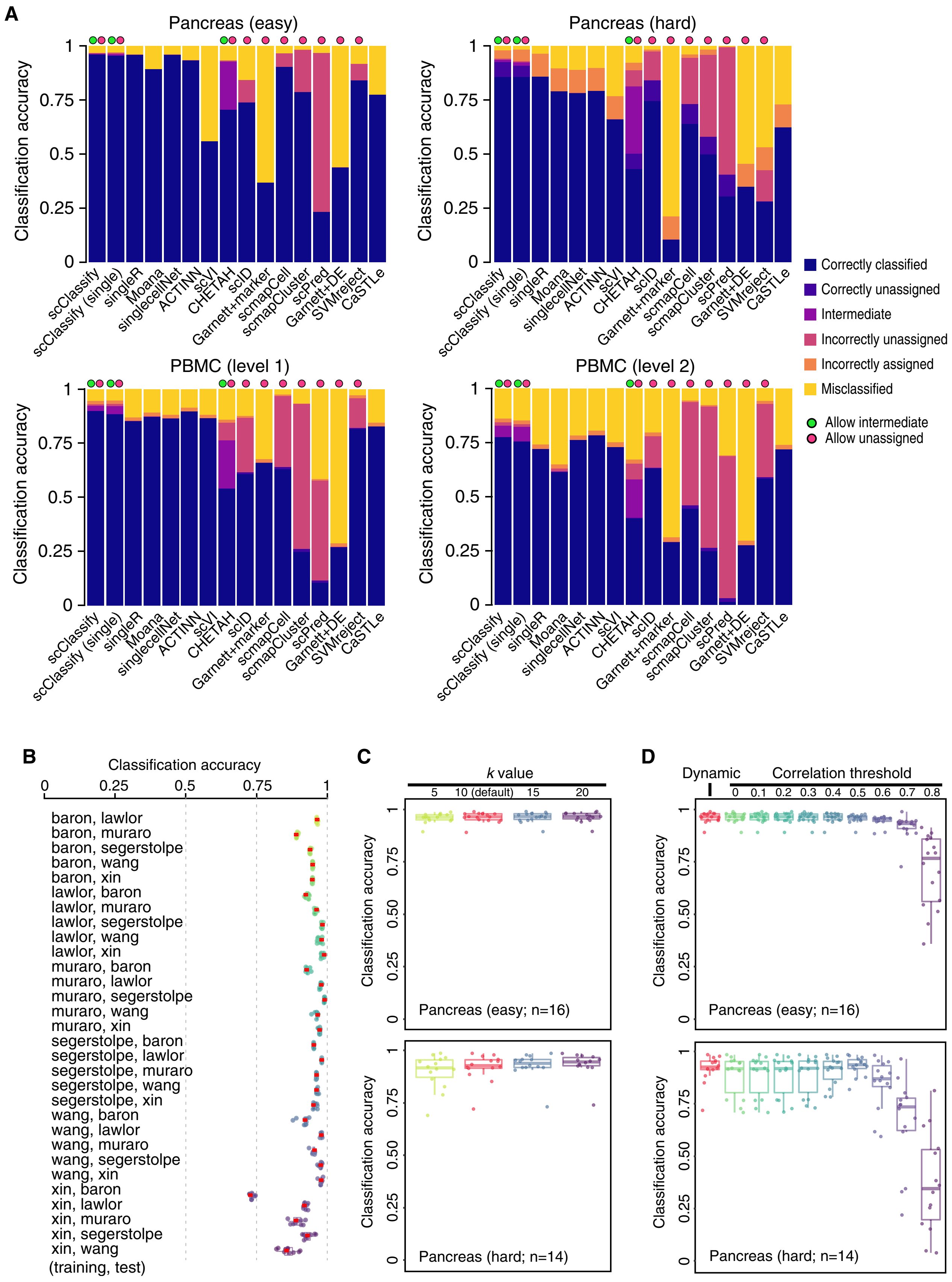


**Figure EV2.**



© 2020 The Authors

**Figure EV3. Sample size estimation results. Related to Fig 3.**

A A 2-by-2 panel of collections of boxplots demonstrating the validation of the sample size calculation using the PBMC10k dataset. The *x*-axis indicates the sample size (*N*), and the *y*-axis indicates the accuracy rate. The left panel indicates the results for the pilot data (20% of the full dataset), and the right panel indicates the results for the reference–test data (the remaining 80% data), representing the data that would be obtained in a follow-up experiment. The top panel indicates the results of predicting PBMC at the top level of the cell type tree, while the bottom panel indicates the results of cell type prediction at the second level of the cell type tree. Each boxplot ranges from the first to third quartile of classification accuracy with the median as the horizontal line. The lower and higher whiskers of boxplot are extended to the first quartile minus 1.5 interquartile range and the third quartile plus 1.5 interquartile, respectively.

B The fitted learning curves on the same data where red solid lines indicate the learning curves by fitting mean accuracy rate of pilot data; red dashed lines are the learning curves obtained by fitting the learning curves to the upper (75%) and lower (25%) quartile of accuracy rate of pilot data. The blue lines indicate the learning curves by fitting the mean of the accuracy rate for the follow-up reference and test dataset.

C Down-sampling of the PBMC10k data using DECENT's beta-binomial capture model (Ye *et al*, 2019). Boxplots indicate the accuracy rates of the cell predictions from down-sampled data for the top (red) and second level (blue) of the cell type tree. Each boxplot ranges from the first to third quartile of classification accuracy with the median as the horizontal line. The lower and higher whiskers of boxplot are extended to the first quartile minus 1.5 interquartile range and the third quartile plus 1.5 interquartile, respectively. The *x*-axis indicates the down-sampling parameter of the beta-binomial distribution (that is, the ratio of capture efficiency in the down-sampled dataset relative to the original dataset), and the *y*-axis denotes the accuracy rate.

© 2020 The Authors

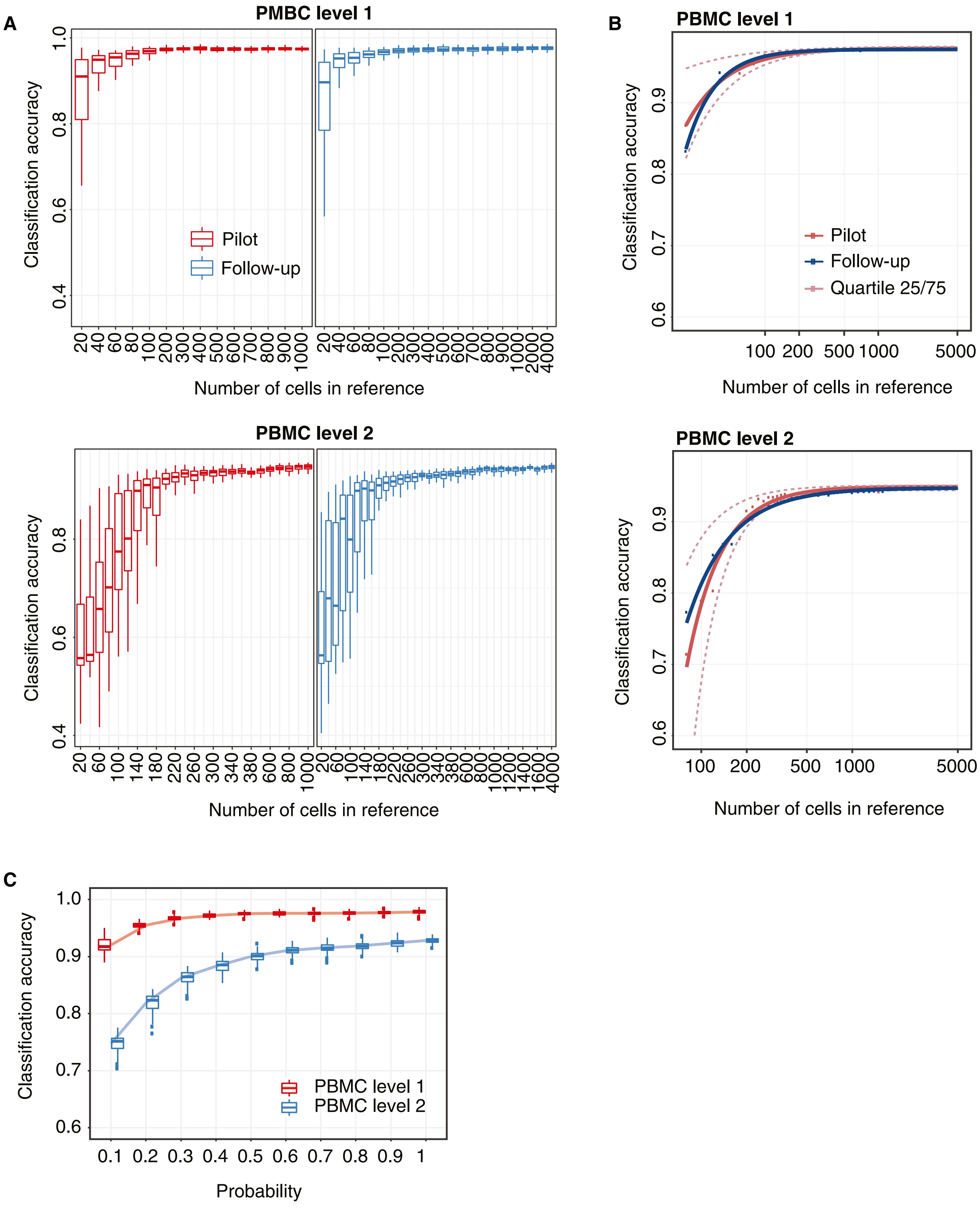


**Figure EV3.**

 © 2020 The Authors

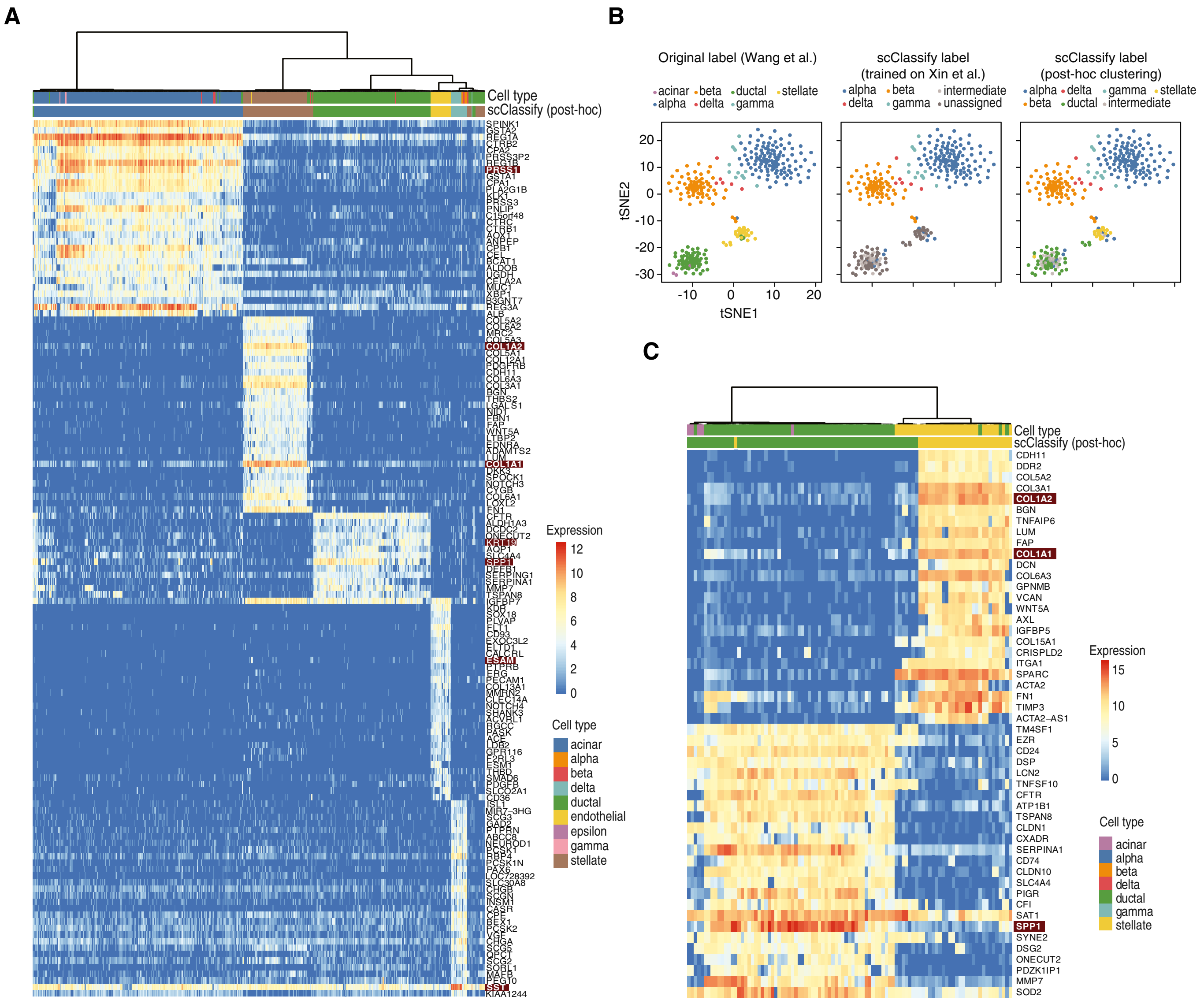


**Figure EV4. *Post hoc* clustering and validation by marker genes. Related to Fig 4.**

A Heatmap of the top 20 differentially expressed genes from each of the five cell type clusters generated through *post hoc* clustering of the Xin-Muraro data pair. Here, Xin *et al* data are used as the reference dataset and Muraro *et al* data as the query dataset. The heatmap is coloured by the log-transformed expression values. The red rectangles indicate markers that are consistent with those found in the original study.

B A 1-by-3 panel of tSNE plots of Wang *et al* from the human pancreas data collection colour-coded by original cell types given in Wang *et al* (2016) (left panel), the scClassify label generated using Xin *et al* as the reference dataset (middle panel) and the scClassify predicted cell types after performing *post hoc* clustering (right panel).

C Heatmap of the top 20 differentially expressed genes from each of the two cell type clusters generated from *post hoc* clustering of the Xin-Wang data pair. The heatmap is colour-coded by the log-transformed expression level. The red rectangles indicate markers that are consistent with those found in the original study.

© 2020 The Authors
